# CD34-selected versus unmanipulated autologous haematopoietic stem cell transplantation in the treatment of severe systemic sclerosis: a post hoc analysis of a phase I/II clinical trial conducted in Japan

**DOI:** 10.1186/s13075-019-1823-0

**Published:** 2019-01-22

**Authors:** Masahiro Ayano, Hiroshi Tsukamoto, Hiroki Mitoma, Yasutaka Kimoto, Mitsuteru Akahoshi, Yojiro Arinobu, Toshihiro Miyamoto, Takahiko Horiuchi, Hiroaki Niiro, Koji Nagafuji, Mine Harada, Koichi Akashi

**Affiliations:** 1 Department of Medicine and Biosystemic Science, Kyushu University Graduate School of Medical Sciences, 3-1-1 Maidashi, Higashi-ku, Fukuoka, 812-8582 Japan; 2 Department of Cancer Stem Cell Research, Kyushu University Graduate School of Medical Sciences, 3-1-1 Maidashi, Higashi-ku, Fukuoka, 812-8582 Japan; 3 Department of Internal Medicine, Kyushu University Beppu Hospital, 4546 Tsurumibaru, Tsurumi, Beppu, 874-0838 Japan; 4 Department of Medical Education, Kyushu University Graduate School of Medical Sciences, 3-1-1 Maidashi, Higashi-ku, Fukuoka, 812-8582 Japan; 5 Present Address: Department of Rheumatology, Shin-Kokura Hospital, 1-3-1 Kanada, Kokurakita-ku, Kitakyushu, 803-8505 Japan; 6 Present Address: Division of Hematology and Oncology, Department of Medicine, Kurume University School of Medicine, 67 Asahi-machi, Kurume, 830-0011 Japan; 7 Present Address: Medical Center for Karatsu-Higashimatsuura Medical Association, 2566-11 Chiyoda-machi, Karatsu, 847-0041 Japan

**Keywords:** Systemic sclerosis, Scleroderma, Haematopoietic stem cell transplantation, CD34

## Abstract

**Background:**

The effectiveness of autologous haematopoietic stem cell transplantation (auto-HSCT) in treating severe systemic sclerosis (SSc) is established; however, the necessity of purified CD34+ cell grafts and the appropriate conditioning regimen remain unclear. This study aimed to compare the efficacy and safety of CD34-selected auto-HSCT with unmanipulated auto-HSCT to treat severe SSc.

**Methods:**

This study was a post hoc analysis of a phase I/II clinical trial conducted in Japan. Nineteen patients with severe SSc were enrolled. Peripheral blood stem cells (PBSCs) were mobilised with cyclophosphamide (4 g/m^2^) and filgrastim (10 μg/kg/day). Following PBSC collection by apheresis, CD34+ cells were immunologically selected in 11 patients. All patients were treated with high-dose cyclophosphamide (200 mg/kg) monotherapy as a conditioning regimen and received CD34-selected (*n* = 11) or unmanipulated auto-HSCT (*n* = 8). Changes in skin sclerosis and pulmonary function were assessed over an 8-year follow-up period. Differences in the changes, toxicity, progression-free survival (PFS) and overall survival were compared between patients who had received CD34-selected auto-HSCT and those who had received unmanipulated auto-HSCT.

**Results:**

Skin sclerosis progressively improved after transplantation over an 8-year follow-up period in both groups, and the improvement was significantly greater in the CD34-selected group than in the unmanipulated group. Forced vital capacity in the CD34-selected group continuously increased over 8 years, whereas in the unmanipulated group it returned to baseline 3 years after transplantation. Toxicity and viral infections, such as cytomegalovirus infection and herpes zoster, were more frequently found in the CD34-selected group than in the unmanipulated group. The frequency of severe adverse events, such as bacterial infections or organ toxicity, was similar between the two groups. No treatment-related deaths occurred in either treatment group. PFS of the CD34-selected group was greater than that of the unmanipulated group, and the 5-year PFS rates of the CD34-selected and unmanipulated group were 81.8% and 50% respectively.

**Conclusions:**

CD34-selected auto-HSCT may produce favourable effects on improvement of skin sclerosis and pulmonary function compared with unmanipulated auto-HSCT. Use of CD34-selected auto-HSCT with high-dose cyclophosphamide monotherapy as a conditioning regimen may offer an excellent benefit-to-risk balance.

**Electronic supplementary material:**

The online version of this article (10.1186/s13075-019-1823-0) contains supplementary material, which is available to authorized users.

## Background

Systemic sclerosis (SSc) is an autoimmune disease characterised by vascular damage and fibrosis of the skin and internal organs [1, 2]. Although immune dysfunction and inflammation play important roles in SSc pathogenesis [3], the efficacy of immunosuppressive therapy, such as corticosteroids or cyclophosphamide, for SSc patients is limited [4, 5]. Therefore, SSc patients with severe skin sclerosis and/or major organ involvement continue to have a poor prognosis, with a 5-year estimated survival rate of approximately 50% [6, 7].

Autologous haematopoietic stem cell transplantation (auto-HSCT) was introduced for the treatment of SSc in 1996; since then, an increasing number of SSc patients resistant to conventional therapy have been treated using this method [8]. Many trials of auto-HSCT for severe SSc have demonstrated improvement of skin sclerosis and stabilisation or improvement of pulmonary function [9–15]. More recently, three prospective randomised controlled trials (RCTs) have been published: The American Scleroderma Stem Cell versus Immune Suppression Trial (ASSIST), The Autologous Stem Cell Transplantation International Scleroderma (ASTIS) and Scleroderma: Cyclophosphamide or Transplantation (SCOT) [16–18]. The ASTIS and SCOT trials revealed that auto-HSCT for early diffuse cutaneous SSc conferred a significant long-term, event-free survival benefit [17, 18], and all trials showed improved skin sclerosis and forced vital capacity and patient reported outcome measurements with the use of auto-HSCT [16–18].

The effectiveness of auto-HSCT in treating severe SSc is established; however, the necessity of purified CD34+ cell grafts and the appropriate conditioning regimen remain unclear [19–21]. In the present study, we aimed to reveal the influence of purified CD34+ cell grafts on auto-HSCT and compare the efficacy and safety of CD34-selected auto-HSCT with those of the unmanipulated ones. Data were collected from a phase I/II clinical trial conducted in Japan.

## Methods

### Study design and patients

This study was a post hoc analysis of a non-randomised phase I/II clinical trial conducted at a single centre (Kyushu University Hospital) in Japan. Data were obtained from all consecutive patients enrolled in the trial.

The trial was performed to assess the efficacy and safety of auto-HSCT in Japanese patients with severe SSc. Patients were enrolled from 2002 to 2009. The inclusion and exclusion criteria have been previously published [22, 23]. In brief, patients were eligible if they were aged between 16 and 65 years, fulfilled the 1980 classification criteria of the American College of Rheumatology for SSc [24], had moderate-to-severe skin sclerosis [modified Rodnan skin score (mRSS), ≥ 15] that had rapidly developed over the previous 4 years [25] and had one or more of the following: pulmonary, cardiac, or renal involvement. Patients with diffuse cutaneous SSc who had mild skin sclerosis (mRSS < 15) or those with limited cutaneous SSc were considered eligible for inclusion when progressive and life-threatening interstitial pneumonia was present. Patients with uncontrolled arrhythmia, severe heart failure, pulmonary hypertension, severe respiratory failure, and renal failure were excluded from the study.

The study was approved by the ethics committee of Kyushu University Hospital and conducted according to the Declaration of Helsinki. Written informed consent was obtained from all patients.

### Procedures

CD34-selected auto-HSCT was performed in 2002–2007 and unmanipulated auto-HSCT in 2007–2009, according to the time that patients were enrolled. Peripheral blood stem cells (PBSCs) were mobilised during haematologic recovery after a relatively high dose of cyclophosphamide (2 g/m^2^) for 2 days, followed by administration of recombinant human granulocyte-colony stimulating factor (G-CSF, filgrastim, 10 μg/kg/day; Kirin Brewery, Tokyo, Japan). PBSCs were collected by apheresis to obtain 2 × 10^6^ CD34+ cells/kg or more, and then CD34+ cells were positively selected using anti-CD34 immunomagnetic beads (CliniMACS; Miltenyi Biotec, Glandbach, Germany). Conditioning was performed using high-dose cyclophosphamide (50 mg/kg) for 4 days, from day − 5 to − 2 and freeze-thawed CD34+ cells were transplanted on day 0. From 2007 onwards, the positive selection of CD34+ cells was skipped and unmanipulated grafts were infused. None of the patients received anti-thymocyte globulin (ATG).

All patients were followed up for at least 8 years after transplantation. The extent of skin sclerosis measured by mRSS, which was evaluated by the same investigator (H.T.), and pulmonary function were assessed every 6 months in the first year and then every 12 months.

### Outcomes

In this analysis, we compared the efficacy and safety of CD34-selected auto-HSCT with those of unmanipulated auto-HSCT. The primary efficacy endpoint was the change from baseline in mRSS over a period of 8 years. The secondary efficacy endpoints were change in pulmonary function, forced vital capacity (FVC) and diffusing capacity of carbon monoxide (DLCO), over 8 years, progression-free survival (PFS) and overall survival. The safety endpoints were the frequency of treatment-related deaths and toxicity. PFS was defined as the time in days from transplantation until death (by any cause) or progression or new occurrence of organ involvement. Toxicity was assessed using the National Cancer Institute Common Toxicity Criteria.

### Immunological analysis

Heparinised whole blood was stained with the following directly conjugated monoclonal antibodies: anti-CD3-fluorescein isothiocyanate (FITC), anti-CD4-FITC, anti-CD8-phycoerythrin (PE) and anti-CD19-PE (eBioscience, San Diego, CA, USA). Following lysis, the cells were analysed using a BD FACSCanto flow cytometer (BD Biosciences, San Jose, CA, USA). Results were expressed as the absolute number of cells.

### Statistical analysis

Continuous variables were summarised by mean ± standard deviation (SD) or median with an interquartile range. Categorical variables were presented as frequencies and percentages. The differences between two groups were analysed using Student’s *t* test for normally distributed continuous variables, Mann–Whitney *U* test for non-normally distributed variables and Fisher’s exact test for categorical variables. Comparison of the changes between two groups was conducted by a linear mixed-effect model; this model included the fixed effects of treatment, month after auto-HSCT and treatment × month interaction, as well as the random intercept for patient and random slope for month. Survival was analysed using Kaplan–Meier survival curves, and log-rank statistics were used for group comparison. All tests were two-tailed, and *p* values < 0.05 were considered significant. All analyses were performed using STATA version 14.0 (StataCorp, Texas, USA).

## Results

### Patients

Nineteen patients were enrolled. The mean ± SD age of the patients was 53.7 ± 6.9 years and 14 patients were females. In this non-randomised trial, 11 patients from 2002 to 2006 received CD34-selected auto-HSCT and 8 patients from 2007 to 2009 received unmanipulated auto-HSCT. As described in Table 1, the baseline clinical characteristics of the patients were generally similar between the two treatment groups, with the exception of FVC. FVC of the patients in the CD34-selected group was lower than in the unmanipulated group, mainly because patients without interstitial pneumonia were only included in the unmanipulated group.

**Table 1:** Baseline clinical characteristics of patients with systemic sclerosis

	CD34-selected (*n* = 11)	Unmanipulated (*n* = 8)	*p* value
Age, years	52.3 ± 7.9	55.8 ± 5.0	0.29
Female, *n* (%)	8 (73)	7 (88)	0.60
Smokers, *n* (%)	4 (36)	4 (50)	0.66
Disease duration, median (IQR), years	1.4 (1.2–2.8)	1.2 (0.9–1.7)	0.18
DcSSc with severe skin sclerosis, *n* (%)	9 (82)	6 (75)	1.00
Modified Rodnan skin score	21.5 ± 9.6	22.9 ± 14.0	0.81
Interstitial pneumonia, *n* (%)	11 (100)	6 (75)	0.16
Percent predicted FVC	63.1 ± 14.7	83.1 ± 17.3	0.01
Percent predicted DLCO	46.4 ± 17.1	49.8 ± 22.9	0.71
LVEF	70.1 ± 8.0	74.5 ± 6.8	0.29
Anti-Scl70 antibody positive, *n* (%)	9 (82)	4 (50)	0.32
Previous treatment, *n* (%)	10 (91)	5 (62.5)	0.26
Corticosteroids, *n* (%)	10 (91)	5 (62.5)	0.26
Cyclophosphamide, *n* (%)	4 (36)	3 (38)	1.00
Tacrolimus or cyclosporine, *n* (%)	3 (27)	2 (35)	1.00

Data are presented as mean ± standard deviation unless otherwise indicated

*IQR* interquartile range, *FVC* forced vital capacity, *DLCO* diffusing capacity of carbon monoxide, *LVEF* left ventricular ejection fraction

### Infused grafts, haematopoietic recovery and immune reconstitution

The numbers of infused CD34+ cells were similar between the two groups, whereas the number of infused CD3+ cells in the unmanipulated group was almost 4000 times more than that in the CD34-selected group (Table 2). All patients achieved rapid haematopoietic engraftment, and the time for neutrophil and platelet engraftment was not markedly different between the two groups (Table 2). In terms of reconstitution of CD4+ and CD8+ T cells and CD19+ B cells, the absolute numbers of each subpopulation showed no clear differences between the two groups during a 5-year follow-up period, although the infused lymphocytes were very different (Additional file 1: Figure S1).

**Table 2:** Number of infused cells and time to haematopoietic recovery

	CD34-selected (*n* = 11)	Unmanipulated (*n* = 8)	*p* value
CD34+ cells, median (IQR), ×10^6^ cells/kg	4.2 (2.2–7.2)	3.5 (2.1–7.8)	0.77
CD3+ cells, ×10^6^ cells/kg	0.01 (0.01)	45.6 (28.1)	0.002
Neutrophil > 0.5 × 10^9^ cells/l, day	12.3 (2.6)	13.8 (2.2)	0.23
Platelet > 50 × 10^9^ cells/l, day	13.3 (3.2)	12.8 (3.0)	0.73

Data are presented as mean ± standard deviation unless otherwise indicated

*IQR* interquartile range

### Skin sclerosis

**Fig. 1:**
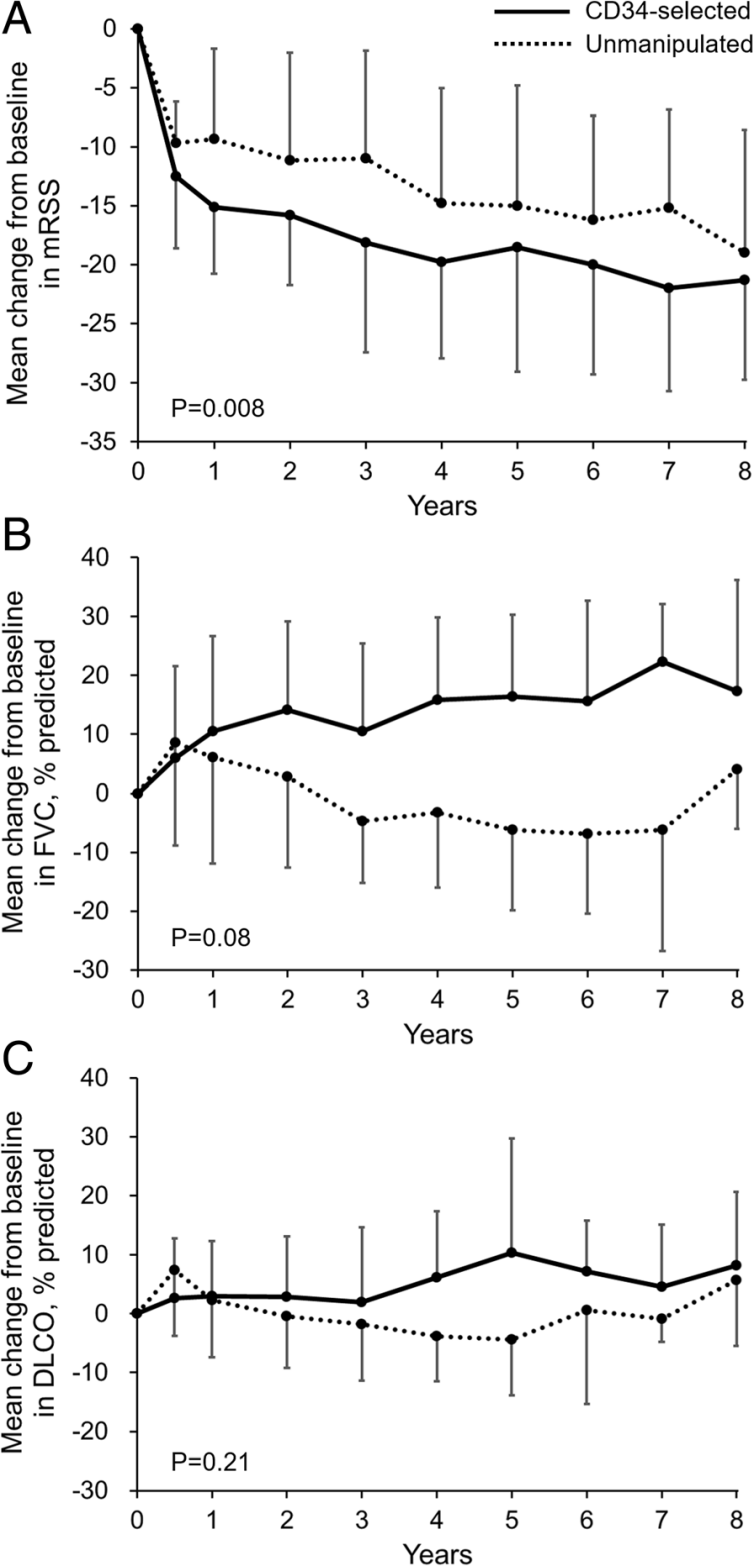
Changes from baseline in skin sclerosis and pulmonary function over an 8-year period. Mean changes from baseline in the modified Rodnan skin score (mRSS; shown in **a**), percent predicted forced vital capacity (FVC; shown in **b**) and diffusing capacity of carbon monoxide (DLCO; shown in **c**) over an 8-year period. Results from the CD34-selected group and the unmanipulated group were compared. In the analysis of mRSS, patients with moderate-to-severe skin sclerosis (mRSS, ≥ 15) were included (CD34-selected, *n* = 9; unmanipulated, *n* = 6). Error bars represent standard deviation

Skin sclerosis progressively improved after transplantation in both groups. The mean ± SD mRSS was markedly decreased from 26.7 ± 7.3 to 9.1 ± 6.0 at 5-year post-transplant, and the improvement was maintained during follow-up in the patients with moderate-to-severe skin sclerosis (Additional file 1: Figure S2A). It was interesting to note that the change from baseline of mRSS was significantly greater in the CD34-selected group than in the unmanipulated group (Fig. 1a).

### Pulmonary function

Pulmonary function was assessed by FVC and DLCO over an 8-year follow-up period. FVC was gradually improved after transplantation, and the mean ± SD score of percent predicted FVC was increased from 71.5% ± 18.4% to 82.1% ± 18.7% and 84.8% ± 22.9% at 5-year and 8-year post-transplant, respectively (Additional file 1: Figure S2B). In contrast, DLCO remained stable over the 8-year follow-up period (Additional file 1: Figure S2C). FVC in the CD34-selected group was continuously increased over the 8 years, while that in the unmanipulated group was transiently increased at 6 months but returned to baseline 36 months after transplantation (Fig. 1b). An analysis comparing the CD34-selected and unmanipulated groups demonstrated that the increase in FVC in the CD34-selected group tended to be superior to that of the unmanipulated group. The change in DLCO was similar between both groups (Fig. 1c).

### Survival

**Fig. 2:**
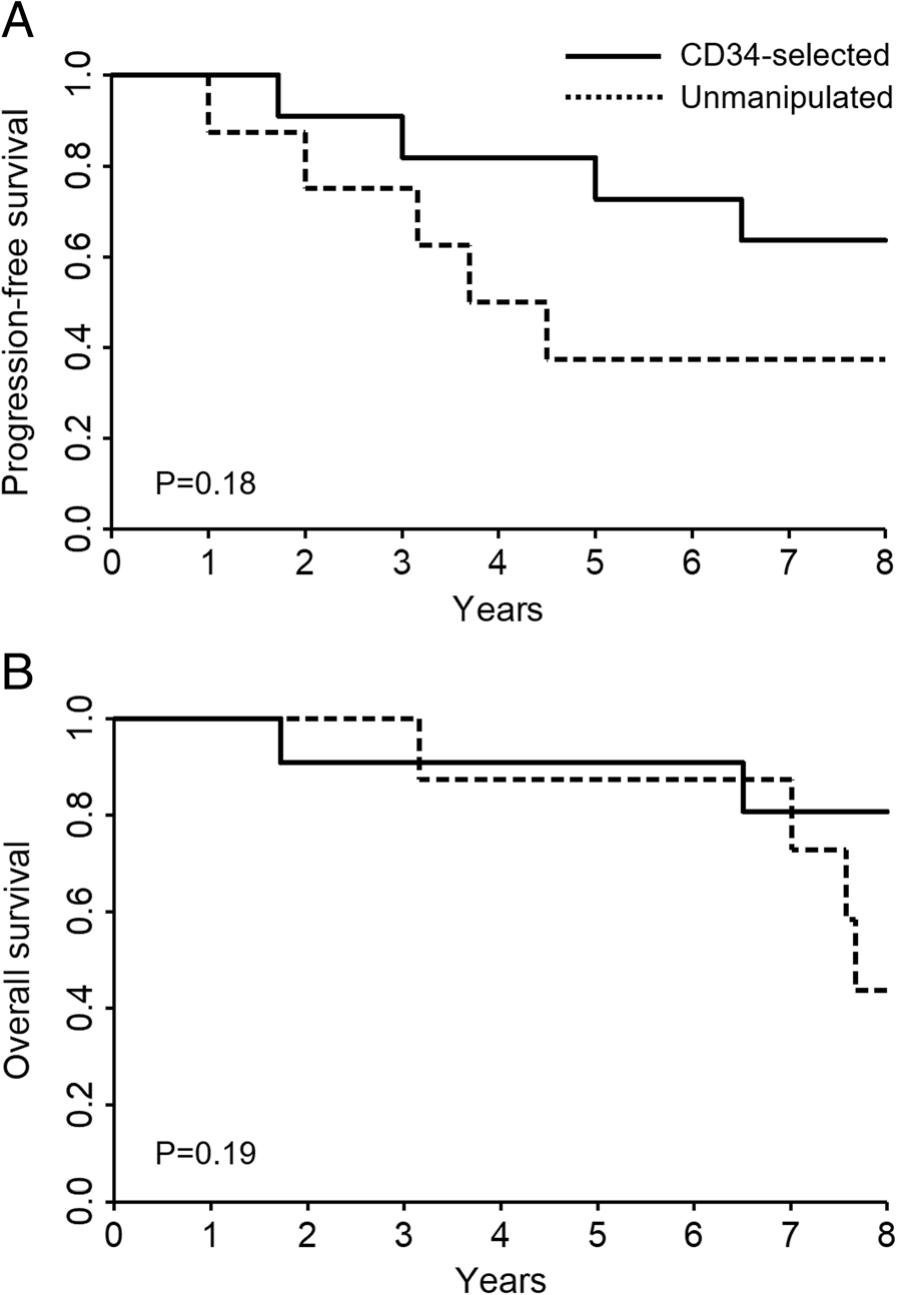
Progression-free survival (**a**) and overall survival (**b**) in the CD34-selected group and the unmanipulated group

No treatment-related deaths occurred in either treatment group. The 5-year and 8-year PFS rates of all patients were 68.4% and 51.3%, respectively. A total of nine events occurred during the observation period of 8 years: four (two deaths, one progression and one occurrence) in the CD34-selected group and five (two deaths, one progression and two occurrences) in the unmanipulated group (Table 3). PFS of the CD34-selected group was greater than that of the unmanipulated group, and the 5-year PFS rates were 81.8% and 50%, respectively (Fig. 2a).

**Table 3:** Deaths and progression or newly occurrence of organ involvement during an 8-year follow-up period

	CD34-selected (*n* = 11)	Unmanipulated (*n* = 8)
Cause of death		
Progression of IP	1	2
Aspiration pneumoniae	1	1
Bacterial pneumoniae	1	0
Intestinal perforation	0	1
Progression		
IP	1	1
New occurrence		
Pulmonary hypertension	1	0
Renal crisis	0	1
Pneumatosis intestinalis	0	1

Data are presented as *n*

*IP* interstitial pneumonia

The 5-year and 8-year overall survival rates of all patients were 89.5% and 65.6%, respectively. Two of the patients in the unmanipulated group died after progression or new occurrence of organ involvement; therefore, the late overall survival of this group decreased (Fig. 2b).

### Toxicity

Viral infections were found more frequently in the CD34-selected group than in the unmanipulated group (CD34-selected, 9/11 patients; unmanipulated, 2/8 patients; *p* = 0.02; Table 4). Cytomegalovirus infection was the most common viral infection observed in both groups, whereas adenovirus haemorrhage cystitis was only found in the CD34-selected group. Herpes zoster was significantly more common in the CD34-selected group. All patients were successfully treated with or without antiviral drugs. The frequency of grade 3 or 4 adverse events, bacterial infections or organ toxicity was similar between the two groups.

**Table 4:** Adverse events

	CD34-selected (*n* = 11)	Unmanipulated (*n* = 8)	*p* value
Grade 3 or 4 adverse event	6 (55)	2 (25)	0.35
Viral infections	9 (82)	2 (25)	0.02
CMV infection	7 (64)	1 (13)	0.06
Herpes zoster	6 (55)	0 (0)	0.02
Adenovirus haemorrhagic cystitis	2 (18)	0 (0)	0.49
Bacterial pneumonia	1 (9)	0 (0)	1.00
Sepsis	2 (18)	1 (13)	1.00
Cardiovascular	4 (36)	2 (25)	1.00
Pulmonary	1 (9)	1 (13)	1.00
Gastrointestinal haemorrhage	2 (18)	0 (0)	0.49
Hepatic	6 (55)	4 (50)	1.00

Data are presented as *n* (%)

## Discussion

This study indicates that CD34-selected auto-HSCT is superior to unmanipulated auto-HSCT in improving skin sclerosis and pulmonary function, with a minimum increase in serious complications, for the treatment of SSc.

Although there have been many studies on the effectiveness of auto-HSCT in the treatment of severe SSc, the necessity of purified CD34+ cell grafts remained unclear [21]. In this clinical trial, we performed unmanipulated auto-HSCT from 2007 to 2009; this was based on similar outcomes of efficacy and safety reported in patients with rheumatoid arthritis undergoing unmanipulated and CD34-selected auto-HSCT [26]. According to the most recent analysis using the European registry for SSc, there was no difference in the overall survival, incidence of relapse or disease progression of the patients who received either unmanipulated or CD34-selected grafts [27]. However, the effectiveness of such grafts on skin sclerosis and pulmonary function remained unknown. Here, the results of this study show that improvements in skin sclerosis and pulmonary function due to CD34-selected auto-HSCT are maintained for at least 8 years, which is superior to results achieved with unmanipulated auto-HSCT.

Our results demonstrated that both CD34-selected and unmanipulated auto-HSCT are effective in improving skin sclerosis; this is consistent with many previous studies [9–21]. Skin sclerosis was remarkably improved within 6 months, and gradual improvement continued for a period of 8 years; however, CD34-selected auto-HSCT was significantly better at improving skin sclerosis than unmanipulated auto-HSCT. The results of RCTs, ASTIS (CD34-selected auto-HSCT) and ASSIST (unmanipulated auto-HSCT), in which the same conditioning regimen was used, demonstrated improvements in skin score of − 79% and − 45%, respectively, over a 2-year period [16, 17]. Given that there are differences in patient characteristics and the conditioning regimen used, the results cannot be subjected to a simple comparison; however, the results of the present study (CD34-selected, − 62%; unmanipulated, − 36%) showed the same trend. The effectiveness of CD34-selected auto-HSCT on skin sclerosis requires verification with an RCT.

The increase in FVC in the CD34-selected group was maintained for a period of 8 years, whereas FVC in the unmanipulated group returned to baseline 3 years after treatment. There have been multiple reports, including RCTs, indicating improvement in pulmonary function over a 2-year period [16–18], but the long-term effectiveness and effect of purified CD34+ cells remain unclear. Although the current study was a non-randomised comparative study and the difference in the baseline data required consideration, this is the first study to show different rates of improvement in FVC over an 8-year period. However, the improvements observed in pulmonary function may have been due to selection bias and characteristics of the conditioning regimen. In this trial, we recruited patients with active and progressive interstitial pneumonia not leading to honeycomb lung and administered high-dose cyclophosphamide monotherapy as a conditioning regimen for transplantation. To reduce the risk of development of lung diseases as a consequence of treatment, T-cell depletion with ATG and total body irradiation (TBI) were not performed. This is because that ATG has a risk of acute lung injury due to the immune response and TBI may cause interstitial pneumonia when the protocol of this study was being created.

There were no treatment-related deaths in the present study; this may have been due to the fact that high-dose cyclophosphamide monotherapy was used as a relatively low-intensity conditioning regimen. Additionally, the study was carried out in close cooperation with a haematology department that had vast experience in the field of transplantation. No differences in the overall survival rates for the CD34-selected and unmanipulated groups were observed, in agreement with a previous study [27]. However, unlike the results demonstrated by Oliveira MC et al. [27], the present study suggested that the CD34-selected group was superior in PFS; this may indicate that unmanipulated auto-HSCT with cyclophosphamide, and without ATG or TBI, is insufficient to prevent disease progression. As the results of the registry study and the present study can only be compared to a certain extent, a multi-institutional RCT would be useful to investigate the necessity of purified CD34+ cells.

In terms of adverse events, there were multiple cases of viral infections such as cytomegalovirus infection and herpes zoster in the CD34-selected group, probably due to the absence of lymphocytes just after the auto-HSCT. However, the frequency of bacterial infections and serious adverse events were the same in both groups. Because our investigation of viral infections indicated that careful monitoring and appropriate therapeutic intervention resulted in sufficient infection control, this profile of adverse events was considered to be tolerable. The fact that ATG was not used in this study may have also contributed to the adverse events reported.

The mechanism of action of auto-HSCT is thought to involve resetting the immune system, not only by removing autoreactive lymphocytes, but also by generating new, self-tolerant, lymphocytes derived from transplanted haematopoietic stem cells [28, 29]. Because T cells were extremely deleted by the positive selection of CD34+ cells as shown in Table 2, use of CD34-selected auto-HSCT has a benefit in that it prevents reinfusion of autoreactive lymphocytes that may be associated with SSc pathogenesis. Previous studies showed that CD62L+CD45RA+CD4+ naive T cells were restored faster in CD34-selected auto-HSCT compared with unmanipulated auto-HSCT [30] and that the thymus-dependent T cell reconstitution after CD34-selected auto-HSCT assessed by T cell receptor rearrangement excision circle analysis was superior to that after unmanipulated auto-HSCT [31]. From this point of view, use of CD34-selected auto-HSCT has an advantage in terms of T cell reconstitution. However, use of CD34-selected auto-HSCT may also have the disadvantage of removing the regulatory lymphocytes controlling SSc and of diminishing immunological memory against pathogens. Although the immune reconstitutions investigated in this study were almost identical between CD34-selected and unmanipulated auto-HSCT, more detailed immunological analysis of autoreactive and regulatory lymphocytes needs to be performed.

This study had several limitations. Firstly, analysis was performed using the results of a single-institution non-randomised study on a small number of subjects. Thus, the results of this study need to be verified in a multi-institutional study with a larger sample size. Secondly, our conditioning regimen differed from that used in previous RCTs conducted in Europe and the USA. Because the conditioning with high-dose cyclophosphamide monotherapy followed by CD34-selected HSCT used in the present study led to equivalent clinical results as those found in previous studies without causing treatment-related deaths, optimisation of the conditioning regimen remains an issue for future studies. Finally, the reason and mechanism for the high degree of clinical efficacy observed in the CD34-selected group remain unknown. Although it is possible that the removal of autoreactive lymphocytes via purification contributed to this superiority, we were not able to verify this and therefore further immunological analysis is required in the future.

## Conclusions

CD34-selected auto-HSCT may lead to increased improvement in skin sclerosis and pulmonary function compared to unmanipulated auto-HSCT. Therefore, use of CD34-selected auto-HSCT with high-dose cyclophosphamide monotherapy as a conditioning regimen may offer an excellent benefit-to-risk balance.

## Additional file

Additional file 1: **Figure S1.** Change in lymphocyte reconstitution over a 5-year period. **Figure S2.** Changes in skin sclerosis and pulmonary function over an 8-year period. (PDF 348 kb)

## Abbreviations

ASSIST: The American Scleroderma Stem Cell versus Immune Suppression Trial
ASTIS: The Autologous Stem Cell Transplantation International Scleroderma
ATG: Anti-thymocyte globulin
auto-HSCT: Autologous haematopoietic stem cell transplantation
DLCO: Diffusing capacity of carbon monoxide
FITC: Fluorescein isothiocyanate
FVC: Forced vital capacity
G-CSF: Granulocyte-colony stimulating factor
mRSS: Modified Rodnan skin score
PBSC: Peripheral blood stem cell
PE: Phycoerythrin
PFS: Progression-free survival
RCT: Randomised controlled trial
SCOT: Scleroderma: Cyclophosphamide or Transplantation
SD: Standard deviation
SSc: Systemic sclerosis
TBI: Total body irradiation
