# Supplementary material for: CD34-selected versus unmanipulated autologous haematopoietic stem cell transplantation in the treatment of severe systemic sclerosis: a post hoc analysis of a phase I/II clinical trial conducted in Japan
Source: Arthritis Res Ther. 2019 Jan 22;21:30. doi: 10.1186/s13075-019-1823-0 (PMC6341635; doi:10.1186/s13075-019-1823-0)
Supplement: Supplementary file 1 — Figure S1. Change in lymphocyte reconstitution over a 5-year period. Figure S2. Changes in skin sclerosis and pulmonary function over an 8-year period. (PDF 348 kb) [file 13075_2019_1823_MOESM1_ESM.pdf]

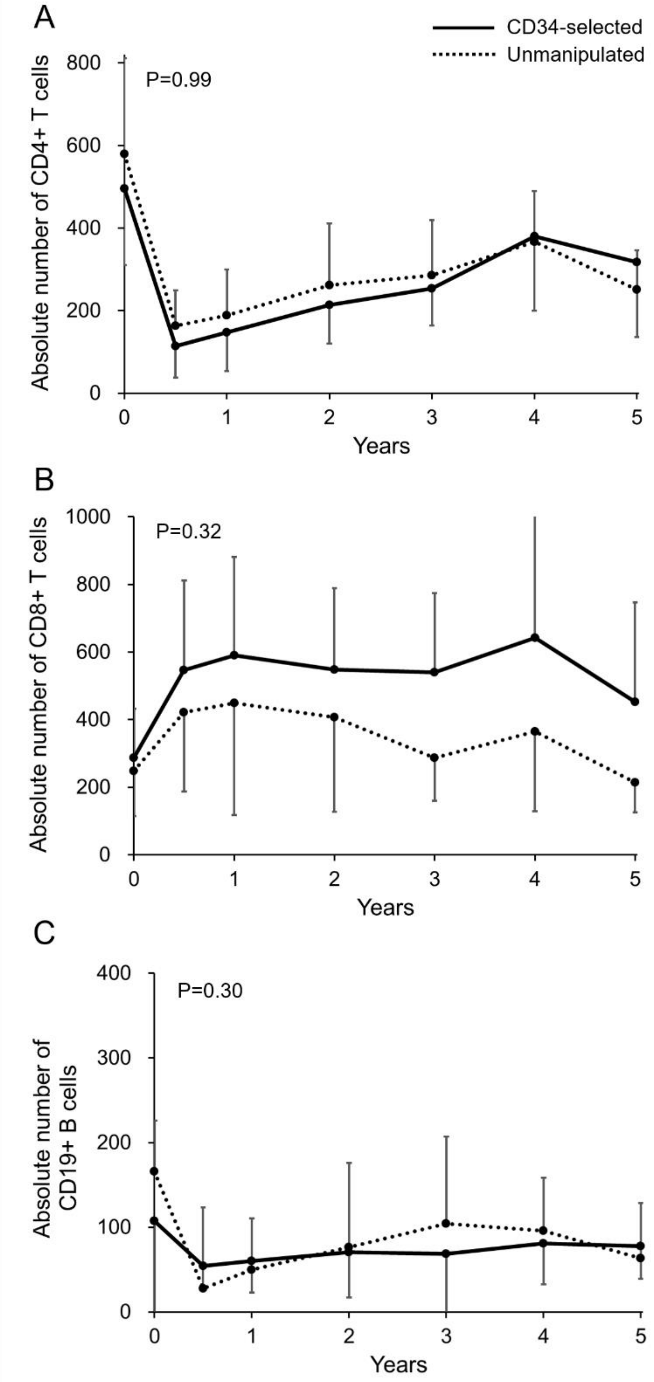

Fig. S1

Mean change in lymphocyte reconstitution over a 5-year period. Absolute numbers of CD4+ T cells (A), CD8+ T cells (B) and CD19+ B cells (C) were compared between the CD34-selected group and the unmanipulated group. Error bars represent standard deviation.

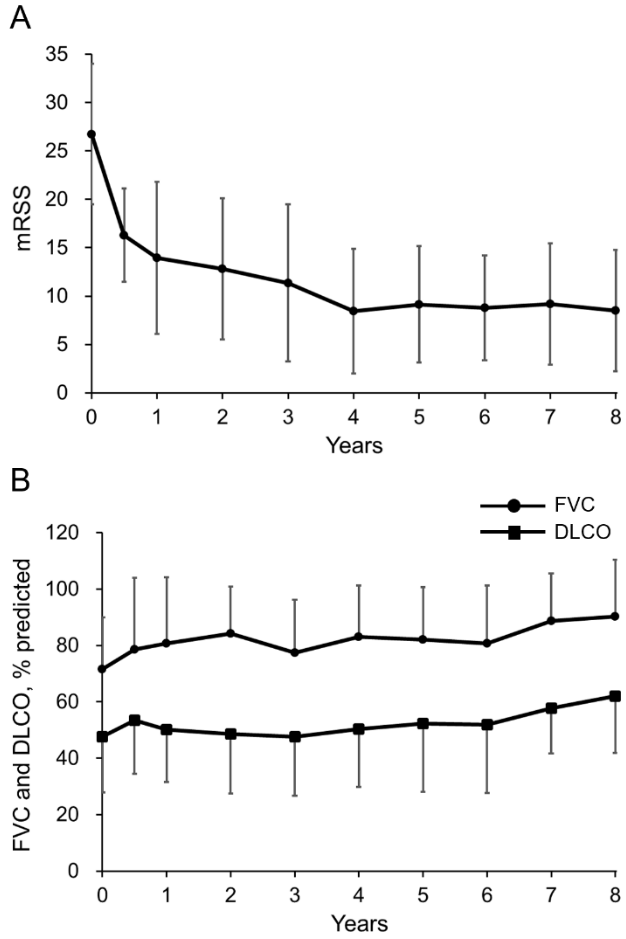

Fig. S2

Mean changes in modified Rodnan skin score (mRSS; shown in A), percent predicted forced vital capacity (FVC; shown in B) and diffusing capacity of carbon monoxide (DLCO; shown in B) over an 8-year period. In the analysis of mRSS, patients with moderate-to-severe skin sclerosis (mRSS, ≥15) were included (n=15). Error bars represent standard deviation.
